# Identification and Quantification of a Pneumococcal Cell Wall Polysaccharide by Antibody-Enhanced Chromatography Assay

**DOI:** 10.3390/vaccines12050469

**Published:** 2024-04-28

**Authors:** James Z. Deng, Zhifeng Chen, James Small, Yue Yuan, Kara Cox, Aimin Tang, Jeanette Roman, Liming Guan, Katrina Feller, Frances Ansbro, Kalpit Vora

**Affiliations:** 1 Vaccine Analytical Research & Development, Merck & Co., Inc., 770 Sumneytown Pike, P.O. Box 4, West Point, PA 19486, USA; 2 Infectious Diseases and Vaccines Research, Merck & Co., Inc., 770 Sumneytown Pike, P.O. Box 4, West Point, PA 19486, USA; zhifeng.chen@merck.com (Z.C.); kara_cox@merck.com (K.C.); aimin_tang@merck.com (A.T.); kalpit.vora@merck.com (K.V.); 3 Analytical Enabling Capabilities, Merck & Co., Inc., 770 Sumneytown Pike, P.O. Box 4, West Point, PA 19486, USA; james_small@merck.com (J.S.); yue.yuan3@merck.com (Y.Y.); 4 Cell Potency Assays, MRL Analytical Research & Development, Merck & Co., Inc., 770 Sumneytown Pike, P.O. Box 4, West Point, PA 19486, USA; jeanette.roman@merck.com (J.R.); liming_guan@merck.com (L.G.); katrina.fritts@merck.com (K.F.); frances_ansbro@merck.com (F.A.)

**Keywords:** pneumococcal conjugate vaccine, antibody-enhanced high-performance chromatography (AE-HPLC), cell wall polysaccharide, phosphocholine-substituted polysaccharide

## Abstract

Multivalent pneumococcal vaccines have been developed successfully to combat invasive pneumococcal diseases (IPD) and reduce the associated healthcare burden. These vaccines employ pneumococcal capsular polysaccharides (PnPs), either conjugated or unconjugated, as antigens to provide serotype-specific protection. Pneumococcal capsular polysaccharides used for vaccine often contain residual levels of cell wall polysaccharides (C-Ps), which can generate a non-serotype specific immune response and complicate the desired serotype-specific immunity. Therefore, the C-P level in a pneumococcal vaccine needs to be controlled in the vaccine process and the anti C-P responses need to be dialed out in clinical assays. Currently, two types of cell-wall polysaccharide structures have been identified: a mono-phosphocholine substituted cell-wall polysaccharide C-Ps1 and a di-phosphocholine substituted C-Ps2 structure. In our effort to develop a next-generation novel pneumococcal conjugate vaccine (PCV), we have generated a monoclonal antibody (mAb) specific to cell-wall polysaccharide C-Ps2 structure. An antibody-enhanced HPLC assay (AE-HPLC) has been established for serotype-specific quantification of pneumococcal polysaccharides in our lab. With the new anti C-Ps2 mAb, we herein extend the AE-HPLC assay to the quantification and identification of C-Ps2 species in pneumococcal polysaccharides used for vaccines.

## 1. Introduction

*Streptococcus pneumoniae* (*S. pneumonia*) bacterial infection can cause invasive pneumococcal diseases (IPDs), resulting in substantial morbidity and mortality worldwide [1]. There are more than 100 known streptococcus pneumoniae serotypes (STs). Among them, more than thirty serotypes can cause IPD. Each pneumococcal serotype contains a unique pneumococcal capsular polysaccharide (PnPs) as its main virulent component [2]. Vaccine development has employed these PnPs as an antigen to provide antigen-specific protection against invasive serotypes included in the vaccine. Initially, multivalent pneumococcal polysaccharide vaccines (PPSVs), such as our polyvalent PNEUMOVAX 23, were developed to combat pneumococcal diseases and worked well in the adult population. However, the PPSVs do not provide enough protection to infants whose immune systems are not fully developed [3,4]. Later, multivalent pneumococcal conjugate vaccines (PCVs) have been developed as effective prophylactic weapons against various invasive pneumococcal serotypes (STs) for both adult and pediatric populations. Recently, a 20-valent PCV, i.e., PREVNAR 20 and a 15-valent PCV, i.e., VAXNEUVANCE were approved for use in infants and elderly people. In both vaccines, pneumococcal polysaccharides are conjugated to a mutant diphtheria toxin protein CRM197 (cross-reactive material 197) to generate robust and long-lasting immune responses [5,6,7].

Besides capsular polysaccharides, cell wall polysaccharides (C-Ps or teichoic acid) are also important components of the *S. pneumoniae* bacterial outer membrane [8,9]. In some cases, C-Ps is an essential co-factor for inducing invasive pneumococcal diseases [10]. C-Ps share the same biosynthesis pathway with PnPs and can covalently link to PnPs through peptidoglycan and/or link to cell wall lipids [11,12,13]. The covalent linkages and the similarity of physicochemical properties to PnPs make the clearance of C-Ps from PnPs in vaccine production very challenging. Therefore, PnPs used in vaccine often contain some residual amount of C-Ps. To develop our 15-valent pneumococcal conjugate vaccine (PCV15), which consists of CRM197 conjugated polysaccharides from the following STs: 1, 3, 4, 5, 6A, 6B, 7F, 9V, 14, 18C, 19A, 19F, 22F, 23F, and 33F [5,6], analytical methods that can detect and quantify C-Ps species in vaccine product and process intermediates are required to control vaccine purity, safety and efficacy.

**Figure 1:**
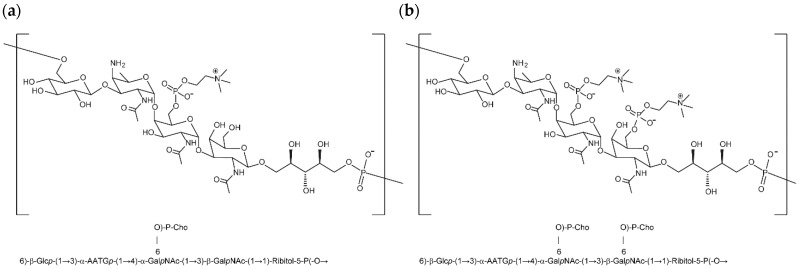
Repeating-unit structures of pneumococcal cell wall polysaccharides. (**a**). C-Ps1 structure: a phosphocholine side chain connects to one GalNAc group (left side) in a C-Ps repeating unit (mono-phosphocholine in each repeating unit). (**b**). C-Ps2 structure: each GalNAc group in a C-Ps repeating unit is substituted by a phosphocholine side chain (di-phosphocholine substitution in each repeating unit).

C-Ps from different pneumococcal serotypes (STs) generally have a conserved core repeating-unit sugar structure composed of tetra saccharides with a glucose (Glc), an AATGal and two N-acetylgalactosamines (GalNac), plus a ribitol phosphate. Only in C-Ps from serotype 5 (ST-5), was an isomeric Glc-to-Gal switch in C-Ps2 reported [14]. Two pneumococcal C-Ps structures have been identified as C-Ps1 and C-Ps2 [15,16,17,18,19,20]. C-Ps2 have two phosphocholine side-chains that occupied both GalNAcs in the repeating unit, whereas C-Ps1 has only one phosphocholine attached to the GalNAc adjacent to a 2-acetoamido-4-amino-2,4,6-trideoxy-galactose (AATG) (Figure 1a,b).

The exact levels of C-Ps for each PnPs serotype are varied and dependent upon the cell culture and purification processes. Although C-Ps mostly exists in a residual level in each PnPs serotype, the total C-Ps content in a multivalent vaccine will add up from each serotype, to reach a concentration that could produce significant immune responses. Innate non-serotype specific immune responses were reported from C-Ps dosing (the administration of C-Ps to study subjects) [21,22,23,24]. These non-serotype-specific anti C-Ps responses may complicate vaccine clinical safety and efficacy evaluations [25]. C-Ps-related issues needed to be addressed in vaccine development according to WHO guidance [26]. Therefore, control of C-Ps level is important to assure quality, safety and efficacy of pneumococcal vaccines.

Reports for C-Ps quantification were mostly performed by NMR [27,28,29]. The NMR assays require a relatively large amount of C-Ps (mg scale) in the sample, and may not be suitable for analysis of residual level (sub μg/mL) of C-Ps content in vaccine product. Furthermore, in a modern multivalent PCV, C-Ps co-exists with 15 or more pneumococcal polysaccharide species in both conjugated or unconjugated forms. Deconvolution C-Ps-specific NMR signals from signals from other polysaccharides for quantification is challenging. There was no report of an analytical method that can separate, identify and quantify a cell wall polysaccharide in the presence of multiple pneumococcal polysaccharides.

**Figure 2:**
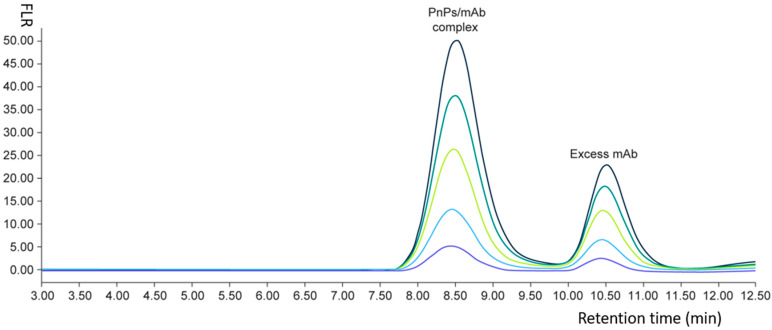
An exemplary AE-HPLC chromatogram for PnPs. PnPs/mAb complex peaks were formed for PnPs at five different concentrations (chromatogram traces with different colors).

Previously, we have established an antibody-enhanced high-performance liquid chromatography (AE-HPLC) assay for identification and serotype-specific quantification of pneumococcal polysaccharides in a PCV15 vaccine [30]. The AE-HPLC leveraged the specific binding complex formed between a polysaccharide serotype and a specific anti serotype antibody. The antibody polysaccharide complex (APC) was separated on HPLC and used as a surrogate for polysaccharide quantifications (Figure 2). We intended to expand the scope of the AE-HPLC assay to pneumococcal C-Ps. From screening of vaccinee memory B cells, a specific anti C-Ps2 mAb is isolated and generated. With this antibody critical reagent in hand, we have applied the AE-HPLC assay to identify and quantify C-Ps2 in commercial pneumococcal polysaccharides. The AE-HPLC assay demonstrates good assay performance for C-Ps2 quantifications and can be easily adapted for quality control (QC) in vaccine development.

The PnPs/mAb complex is separated from excessive mAb reagent on the chromatogram and used as surrogate for PnPs quantification.

## 2. Materials and Methods

### 2.1. Reagents and Materials

Bis-Tris-HCl 1M solution was purchased from Rigaku Reagents, Inc. (Seattle, WA, USA). Sodium chloride 5M solution was bought from Promega corporation (Madison, WI, USA). C-Ps standards CWPS1 (C-Ps1 standard) and CWPS-multi (C-Ps standard containing both C-Ps1 and C-Ps2) were purchased from SSI Diagnostica A/S (Copenhagen, Denmark). Pneumococcal polysaccharides (PnPs) samples (serotypes 4, 6A, 9V, 12F and 19A) were obtained from ATCC (American Type Culture Collection, Manassas, VA, USA).

### 2.2. Antibody Isolation and Generation

The anti C-Ps2 antibody 2D5 was isolated from the peripheral memory B cells of a PCV13 vaccinee and mAb was generated as in the previously published literature [31,32,33]. Briefly, a 19F antigen conjugated to CRM197 was biotinylated for use in flow cytometry. PBMCs (peripheral blood monocytes) of the PCV13 vaccinee were then stained for CD19+CD27+IgG+ and 19F_CRM+ antigen-specific memory B cells, and single sorted into 96-well plates for 2-week culture. The culture supernatants were tested in ELISA reaction against 19F_CRM, and the corresponding single B cell culture hits were then processed with RNA extraction, PCR reactions and Sanger Sequencing.

The PCR reactions are composed of two steps: RT-PCR and nested PCR. Briefly, total RNA was extracted from single-sorted memory B culture with Qiagen RNeasy Micro Kit (Qiagen, Hilden, Germany) following the manufacturer’s instruction. RT-PCR was applied in the amplification of human antibody genes with the Qiagen One-step RT-PCR kit (Qiagen). The RT-PCR reaction condition was the following: 30 min at 50 °C for the reverse transcription (RT) reaction to synthesize the cDNA, and the RT reaction was then stopped by heat treatment at 95 °C for 15 min. The RT reaction was followed by 40 cycles of polymerase chain reaction (PCR) with the following conditions: 30 s at 94 °C for DNA denaturation into single chains, 30 s at 55° C for annealing of DNA templates with primers, 1 min at 72 °C for extension of newly generated DNA strands from primers. Finally, there was a 10 min elongation at 72 °C and the reaction was then held at 4 °C. The RT-PCR products were then used as templates in nested-PCR to amplify antibody variable regions with Invitrogen pfx50 DNA polymerase (Invitrogen, Carlsbad, CA, USA), and the nested-PCR reaction conditions for amplifying the variable regions of heavy chain, kappa light chain and lambda light chain were the same: 2 min at 95 °C, then 10 cycles of PCR reactions. The conditions were the following: 30 s at 94 °C for DNA denaturation, 30 s at 50 °C for annealing of DNA templates with primers, 1 min at 68 °C for DNA strand extension, followed by another 30 cycles of PCR reactions with increased annealing temperature: 30 s at 94 °C for DNA denaturation, 30 s at 60 °C for DNA template annealing with primers, and 1 min at 68 °C for DNA strand extension. In the end, there was a 7 min elongation at 68 °C and the reaction was then held at 4 °C. The nested-PCR products were then agarose gel purified (Qiagen), and applied to the Sanger Sequencing.

The Sanger Sequencing: discovered antibodies with naturally paired heavy- and light-chain sequences were synthesized for CHO-3E7 mammalian-cell transient transfection and expression (GenScript, Piscataway, NJ, USA). After 6 days’ culture, the cell culture supernatants were harvested, and the expressed antibodies were then purified and stored in 1× phosphate buffered saline (PBS) at pH 7.2. The antibody was finally QC tested by SDS-PAGE and Western blot analysis.

### 2.3. Sandwich ELISA for Anti C-Ps mAb Binding to C-Ps-Containing Species

A polysaccharide-specific Sandwich ELISA was employed to screen the anti C-Ps mAb against several PnPs serotypes as described previously [31]. In this ELISA assay, the plates were coated with a CRM capture mAb (1 μg/mL). At 2–8 °C, the coated plates were blocked and incubated overnight with serially diluted target PnPs serotypes. After the plate wash, the anti C-Ps mAb (1 μg/mL) was applied to the plates and they were incubated for one hour. The plates were washed, then incubated for one hour with 1:6000 dilution of donkey anti-human antibody conjugated with alkaline phosphatase (Jackson ImmunoResearch, West Grove, PA, USA) in a solution of assay diluent spiked with normal mouse serum (Jackson ImmunoResearch). The plates were washed, 4-Methylumbelliferyl-phosphate (4-MUP) (Virolabs, Chantilly, VA, USA) was applied, and they were incubated for 45 min. The fluorescence signal was recorded on a SpectraMax M2E spectrophotometer at 360 nm (excitation)/450 nm (emission). Data were collected using SoftMax Pro (Molecular Devices, San Jose, CA, USA).

### 2.4. AE-HPLC Assay and Quantification of C-Ps2 in Pneumococcal Polysaccharides

#### 2.4.1. AE-HPLC Chromatography Conditions

The HPLC system was set on a Waters Alliance (Waters Corporation, Milford, MA, USA) equipped with a quaternary pump, sample manager, column compartment and fluorescence (FLR) detector. The FLR was detected using excitation (*Ex*) wavelength at 280 nm and emission (*Em*) wavelength at 352 nm (*Ex/Em* 280 nm/352 nm). Two Tosoh TSKgel GMPWxL columns (7.8 mm × 30 cm, 13 μm particle size, Tosoh Bioscience, Tokyo, Japan) were connected in tandem and used for the separation of antibody/C-Ps2 complex from the antibody; 10 mM Bis-Tris, 150 mM NaCl, pH 6.8 was used as mobile phase and an isocratic flow rate of 1.0 mL/min was employed. The HPLC autosampler was maintained at a temperature of 8 ± 2 °C. Column temperature was set at 30 °C and HPLC run time was 30 min per injection.

#### 2.4.2. Preparation of C-Ps2 Standard Curves

C-Ps2 standard solutions were prepared by dissolving CWPS-multi (SSI Diagnostica A/S, Hillerød, Denmark) in PBS. The C-Ps2 concentration in the standard stock was determined and calibrated by using C-Ps2 concentration for CWPS-multi NMR samples (Section 2.5). The standard was bound to anti C-Ps2 at five or more concentration levels, as in Table 1. To set up antibody C-Ps2 binding reactions for a standard curve, five volume levels (5, 20, 40, and 80 μL) of standard solution containing 1.53 μg/mL C-Ps2 in water were pipetted into five 300 μL HPLC vials, respectively. A total of 15 μL of 1.00 mg/mL of anti CPs-2 mAb (mAb 2D5) in PBS buffer was added into each HPLC vial, plus the binding buffer (10 mM Bis-Tris, 150 mM NaCl, pH 6.8), to bring the total volume in each HPLC vial to 200 μL. The binding reactions were incubated at ambient temperature for two hours, before being put into the HPLC autosampler (with the temperature at 8 °C) for AE-HPLC analysis. A total of 40 μL of each standard binding reaction was injected in duplicate and separated on the HPLC. The mAb C-Ps2 complex peak areas from the duplicated injections were averaged. The standard curve was plotted using the averaged peak area (detected on the HPLC fluorescence detection channel with *Ex/Em* 280 nm/352 nm) vs. the C-Ps2 concentrations in the binding reactions listed in Table 1. A standard-curve linearity of R^2^ > 0.99 is required to pass the assay criteria for quantification. The standard-curve slope (STD slope) and intercept (STD intercept) were calculated from the standard curve by Excel and used for the C-Ps2 quantification of pneumococcal polysaccharide samples set out in Section 2.4.3.

#### 2.4.3. Quantification of C-Ps2 Levels in Pneumococcal Polysaccharides

Each of the ST-4, ST-6A, ST-9V, ST-12F and ST-19A pneumococcal polysaccharides from ATCC were dissolved in water to give a 100 μg/mL of total polysaccharide (total Ps) stock solution in a 2 mL HPLC vial, respectively. Each PnPs stock solution (100 μg/mL) was added into a 300 μL HPLC vial, followed by the addition of 15 μL of 1.00 mg/mL of anti CPs-2 mAb (mAb 2D5) in PBS buffer, plus binding buffer (10 mM Bis-Tris, 150 mM NaCl, pH 6.8). The total volume for a PnPs sample binding reaction was 200 μL as described in Table 2.

Each PnPs sample binding reaction was incubated at ambient temperature for two hours, before being put into the HPLC autosampler for AE-HPLC quantification. Each binding reaction was injected in duplicate at 40 μL. The mAb C-Ps2 complex peak areas were integrated into Empower 3 software (Waters Corporation, Milford, MA, USA). The averaged peak area from duplicate injections was used to calculate C-Ps2 concentration in the sample, as in Equation (1).

(1) $$\mathrm{Sample}[C\text{-}\mathrm{Ps}2]=(\frac{\mathrm{Peak}\;\mathrm{area}-\mathrm{STD}\;\mathrm{intercept}}{\mathrm{STD}\;\mathrm{slope}})*\mathrm{Dilution}\;\mathrm{factor}$$

Sample [C-Ps2] is the measured C-Ps2 concentration (μg/mL) in a pneumococcal polysaccharide (PnPs) sample; STD intercept is the standard-curve intercept; STD slope is the standard-curve slope; the dilution factor is listed in Table 2 for each serotype.

The measured C-Ps2 concentration ([C-Ps2]) from Equation (1) divided by the total polysaccharide concentration in the sample ([Total Ps], 100 µg/mL) will give the percentage of C-Ps2 (% C-Ps2) in a pneumococcal polysaccharide sample, as in Equation (2). This was used to assess the C-Ps levels in these pneumococcal polysaccharides used to generate vaccines.

(2) $$\%\;C\text{-}\mathrm{Ps}2=\left(\frac{\left[C\text{-}\mathrm{Ps}2\right]}{\left[\mathrm{Total}\;\mathrm{Ps}\right]}\right)*100$$

### 2.5. ^1^H and ^31^P NMR for C-Ps Standards

NMR samples for C-Ps standards were prepared by dissolving C-Ps1 (SSI, CWPS), or a mixture of C-Ps1 and C-Ps2 (SSI, CWPS-Multi) in the Deuterium oxide (D_2_O, 99.96% atom D) containing 0.01% (*v*/*v*) dimethylsulfoxide (DMSO), used as an internal intensity standard and 0.01% (*w*/*v*) DSS-*d*_6_, used as an internal chemical-shift standard. The concentration of the C-Ps standard in the NMR sample is about 5 mg/mL. ^1^H NMR spectra were acquired at 49 °C on a Varian DD2 600 MHz spectrometer with a 5 mm HCN CryoProbe and by running the VnmrJ version 4.2. ^1^H NMR spectra were recorded using a standard one-pulse experiment with 32,768 data points, a spectral width of 10 ppm, and 4 scans. For the quantitative study, relaxation time was set to 57 s to ensure a full recovery of each signal. One-dimensional ^31^P NMR spectra were acquired at 25 °C on the Bruker DRX 700 MHz spectrometer with an HCP Cryoprobe. Suitable signal-to-noise ^31^P spectra were recorded, accumulating approximately 256 scans with 32,768 data points and a spectral width of 75 ppm, weighted with 2.0 Hz line broadening, and were Fourier transformed. The ratio of C-Ps1 and C-Ps2 in the CWPS-Multi was provided by SSI and confirmed by ^31^P NMR (Supplementary Figure S2). Quantitative NMR analysis for C-Ps was performed based on the resonance integration of choline signals at 3.22 ppm and the DMSO signal at 2.71 ppm (Supplementary Figure S3).

## 3. Results

### 3.1. Generation and Characterization of an Anti-C-Ps2 mAb

**Figure 3:**
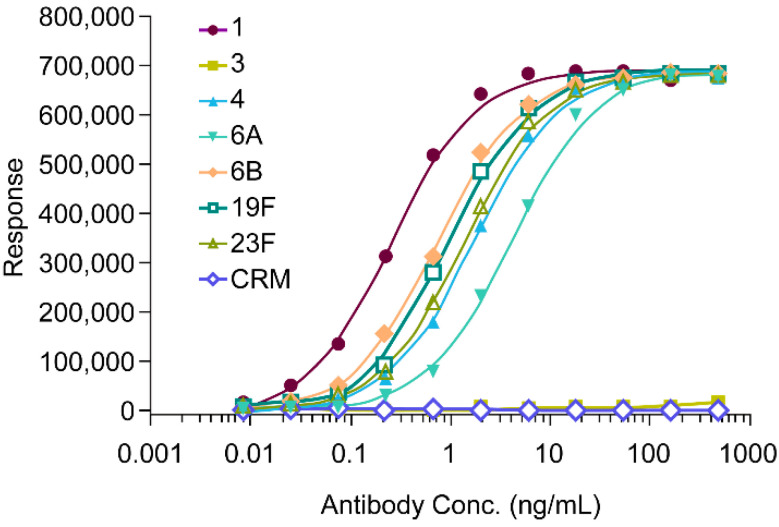
Anti C-Ps mAb binding to C-Ps in PnPs serotypes by ELISA. The anti C-Ps mAb (2D5) binds to C-Ps containing polysaccharide samples from multiple serotypes, but not to the ST-3 sample that lacks C-Ps by NMR. The CRM197 protein (CRM) was used as negative control for mAb binding.

Previously, we have reported the isolation and generation of serotype-specific anti-PnPs antibodies from plasma-blast B cells of a vaccinee [31,32,33]. In a similar effort, in trying to find the ST-19F antibody from memory B cells from the same donor, another interesting monoclonal antibody (mAb) 2D5 capable of binding to PnPs samples from multiple serotypes was identified. The polysaccharide serotypes (ST-1, ST-4, ST-6A, ST-6B, ST-19F and ST-23F) that bind to this mAb all contain residual C-Ps content according to NMR analysis, whereas serotype 3 (ST-3) polysaccharide, which lacks the C-Ps presence did not bind to this mAb in the ELISA assay (Figure 3). The mAb also does not bind to the CRM197 protein (negative control) in ELISA. Therefore, it was proposed that this antibody was an anti C-Ps antibody. Purified C-Ps standards from SSI Diagnostica A/S were used to further evaluate the antibody-binding affinity to the two C-Ps structures, i.e., C-Ps1 and C-Ps2, on AE-HPLC. CWPS1 from SSI Diagnostica A/S standard is known to be pure C-Ps1 (see Supplementary Figure S2 and information from the vendor). It did not show binding affinity to our mAb in the AE-HPLC assay, whereas antibody/C-Ps binding complex was observed on AE-HPLC by using the other SSI C-Ps standard CWPS-multi (Supplementary Figure S1). CWPS-multi contains both purified C-Ps1 and C-Ps2 (see Supplementary Figure S2 and information from the vendor). Therefore, it was concluded that our mAb is bound to C-Ps2 only, but not to the C-Ps1 structure. An AE-HPLC assay using this mAb would be specific for the C-Ps2 species.

### 3.2. AE-HPLC Method Development and Linearity

**Figure 4:**
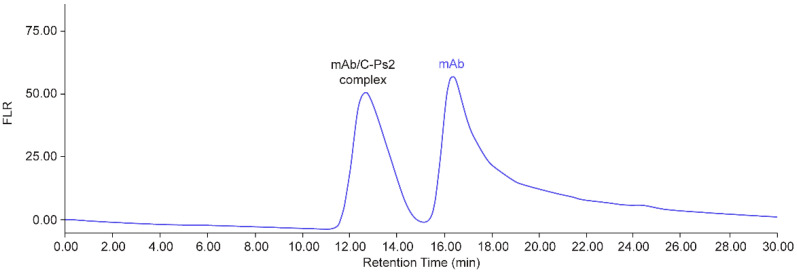
An exemplary AE-HPLC chromatogram for C-Ps2/mAb binding reaction. MAb bound to C-Ps2 formed a complex that is larger than unbound mAb. The mAb/C-Ps2 complex elutes out earlier than the unbound mAb on size-exclusion chromatography.

**Figure 5:**
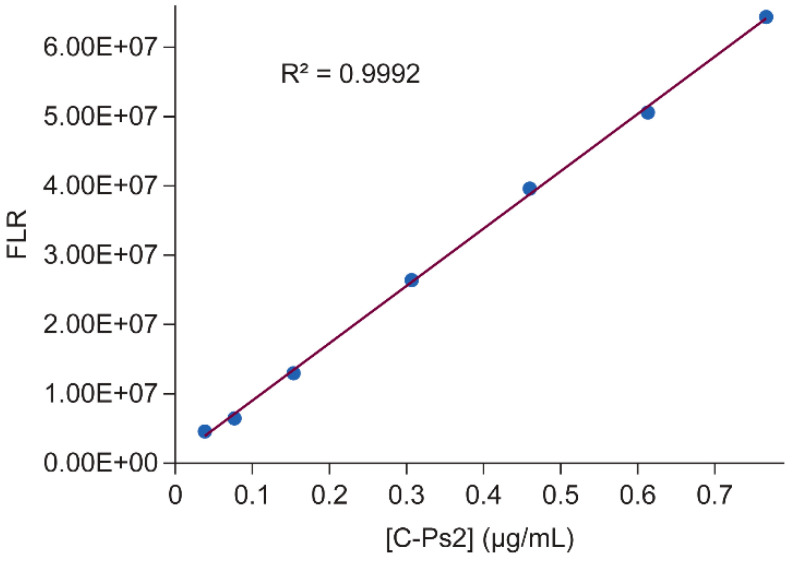
A linear standard curve for C-Ps2 quantification by AE-HPLC assay. Good linearity was observed for mAb/C-Ps2 complex peak area vs. C-Ps2 concentrations.

The size-exclusion chromatography (SEC) method (Section 2.4) was optimized to separate the mAb/C-Ps2 complex from excess mAb (Figure 4), based on column screening and the previous SEC method development experience for pneumococcal vaccines [5,30]. The fluorescence peak area (*Ex/Em* 280 nm/352 nm) from the mAb/C-Ps2 complex was used for quantification. The linearity range of the standard curve was evaluated by binding reactions with excess mAb to C-Ps2 at concentrations from low to high. An excellent linearity (R^2^ > 0.999) was observed with C-Ps2 concentration from ~0.04 to ~0.8 µg/mL (Figure 5, Supplementary Table S1).

### 3.3. Assay Precision and Accuracy

A five-point standard curve within the linearity range was used to evaluate assay precision and accuracy (Table 1). Five pneumococcal polysaccharide serotypes (PnPs) (ST-4, ST-6A, ST-9V, ST-12F and ST-19A) were analyzed for C-Ps2 concentration on the AE-HPLC assay. C-Ps2 was detected in all of these five PnPs (ST-4, ST-6A, ST-9V, ST-12F and ST-19A) with the lowest C-Ps2 level in ST-9V at 0.33% and the highest in ST-4 at 8.9%. Each PnPs serotype was analyzed for six measurements. Good assay precision was observed with %RSD ≤ 11% (*n* = 6) for all serotypes (Table 3, Supplementary Table S2).

To assess the assay accuracy, the C-Ps standard at three different levels was spiked into an ST-19A control sample in the antibody binding reactions. The % recovery of C-Ps2 is within the range of 104% to 107% (Table 4, Supplementary Table S3), representing a good assay accuracy for C-Ps2 quantification. Both assay accuracy and precision have met the assay criteria set by the regulatory agency for chromatography and ligand-binding assays [35]. Therefore, the AE-HPLC is suitable for supporting vaccine development.

## 4. Conclusions and Discussion

An antibody-enhanced HPLC assay (AE-HPLC) has been developed to quantify pneumococcal C-Ps with double phosphocholine sidechain structure (C-Ps2). This represents a novel approach to selectively identify and quantify C-Ps2 species in pneumococcal polysaccharides.

Besides the C-Ps quantification by NMR methods [27,28,29], analytical methods based on hydrolysis of polysaccharides to mono saccharides or small components could also be developed for C-Ps quantification [14,34]. However, complete polysaccharide hydrolysis requires a rather harsh sample treatment, which may lead to poor recovery. NMR can measure samples directly, but it requires amounts measured in milligrams of relatively pure C-Ps material. To quantify the C-Ps level in pneumococcal-polysaccharide or conjugate samples, good resolution of a characteristic C-Ps peak from other polysaccharide peaks and peaks from the sample matrix is required. Also, NMR is not readily adapted in a QC lab.

The AE-HPLC assay is established on automatic chromatography systems and can be easily operated in a QC setting. Compared to NMR, AE-HPLC has much better assay sensitivity and high specificity. These factors allow it to be used for analyzing C-Ps in multivalent-vaccine products in which multiple polysaccharide species are formulated at μg/mL concentrations in complex sample matrices. NMR is a reagent-free assay, whereas AE-HPLC requires the generation of a good anti-C-Ps antibody as a critical reagent. However, once the antibody is generated, it can be used not only on the AE-HPLC platform, but also on other ligand-binding assay platforms, such as ELISA, surface plasmon resonance (SPR) and biolayer interferometry (BLI). C-Ps species in a PCV can exist as conjugate forms with additive components. AE-HPLC-assay specificity is conferred by the antibody specificity that bind to C-Ps epitopes. Therefore, components that conjugate to or are added on to the C-Ps structure or components in the sample buffer should not interfere with assay performance.

Herein, an antibody specific to C-Ps2 was employed. The generation of antibodies that could bind to C-Ps1 or bind to both C-Ps1 and C-Ps2 structures would further expand the application of the AE-HPLC assay and provide more insight into the C-Ps species and their distribution among different pneumococcal serotypes. In summary, an AE-HPLC assay has been developed to quantify residual cell-wall polysaccharides C-Ps2 species in pneumococcal polysaccharides with good linearity, precision and accuracy. The assay performance has met the analytical performance criteria set by regulatory agencies for bioanalytical methods [35,36,37]. Ongoing development of novel antibody reagents would expand the application of this assay to other cell-wall poly-saccharide species and serve as useful tools for vaccine development.

## Figures and Tables

**Table 1:** Preparation of a 5-point C-Ps2 standard curve for AE-HPLC.

Standard	1.53 μg/mL C-Ps2 Stock (µL)	1.00 mg/mL mAb (µL)	Binding Buffer (µL)	Total Vol (µL)	[C-Ps2] (µg/mL)
STD-1	5	15	180	200	0.038
STD-2	20	15	165	200	0.153
STD-3	40	15	145	200	0.306
STD-4	60	15	125	200	0.459
STD-5	80	15	105	200	0.612

**Table 2:** Preparation of C-Ps2 mAb binding reactions for pneumococcal polysaccharide (PnPs) serotypes.

PnPs Serotype (ST)	100 µg/mL PnPs Stock (µL)	1.00 mg/mL mAb (µL)	Binding Buffer (µL)	Total Vol (µL)	Dilution Factor
ST-4	5	15	180	200	40
ST-6A	20	15	165	200	10
ST-9V	100	15	85	200	2
ST-12F	10	15	175	200	20
ST-19A	20	15	165	200	10

**Table 3:** AE-HPLC assay precision.

PnPs Serotype (ST)/Experimental	% C-Ps2 in Each PnPs				
	ST-4	ST-6A	ST-9V	ST-12F	ST-19A
Exp-1	9.0	3.5	0.33	6.1	3.2
Exp-2	8.9	3.4	0.34	6.1	3.2
Exp-3	9.2	3.5	0.35	6.3	3.2
Exp-4	9.1	3.1	0.34	5.7	3.9
Exp-5	8.4	2.9	0.31	5.7	3.9
Exp-6	8.9	3.0	0.32	5.7	4.0
Average (%)	8.9	3.2	0.33	5.9	3.6
%RSD	3.2	9.0	4.0	4.5	11

**Table 4:** Spike recovery at three concentration levels.

Sample	Measured Spike [C-Ps2] (μg/mL)	Theoretical Spike [C-Ps2] (μg/mL)	% Recovery
Spike-1	0.159	0.153	104
Spike-2	0.322	0.307	105
Spike-3	0.572	0.536	107

## Data Availability

The data sets generated and/or analyzed during the current study are available in the article or Supplementary Materials or from the corresponding author on reasonable request.

## Supplementary Materials

The following supporting information can be downloaded at: https://www.mdpi.com/article/10.3390/vaccines12050469/s1. Table S1. Linearity range data for Figure 4. Peak area was averaged from duplicate injections. Table S2. C-Ps2 quantification precision assessment for 5 PnPs sertypes in six runs. Peak area was averaged from duplicate injections. % C-Ps2 was obtained by Sample [C-Ps2] divided by Sample [Total Ps]. Table S3. Preparation of spike sample and calculation for spike recovery. Figure S1. Comparison of mAb binding to SSI CWPS1 (C-Ps1) and mAb binding to SSI CWPS-multi (C-Ps1 + C-Ps2) on AE-HPLC. A. Binding to C-Ps1: no mAb/C-Ps1 complex detected. B. Binding to (C-Ps1 + C-Ps2): mAb/C-Ps2 complex was detected. Figure S2. ^31^P NMR of C-Ps standards: CWPS1 (C-Ps1) and CWPS-multi (C-Ps1 + C-Ps2). One phosphocholine (P-choline) was detected in CWPS1 and two phosphocholines were observed in CWPS-multi. Figure S3. Quantitative NMR analysis for CWPS was performed based on the resonance integration of choline signal at 3.22 ppm and DMSO signal at 2.71 ppm. ^1^H NMR data were accquired at 49 °C in D2O. The concentration of DMSO in the solvent is 1.411 mM. Molecular weight (Da) used in the calculations were 1134 for C-Ps1 repeating unit and 1299 for C-Ps2 repeating unit.

## Abbreviations

AE-HPLC	Antibody-enhanced HPLC assay
Ps	polysaccharides
C-Ps	cell wall polysaccharides
PnPs or CPS	pneumococcal capsular polysaccharides
ST	serotype
PCV	pneumococcal conjugate vaccine
PCV15	15-valent pneumococcal conjugate vaccine
IPD	invasive pneumococcal diseases
WHO	World Health Organization
PBMC	peripheral blood monocytes
SEC	Size-exclusion chromatography
NMR	Nuclear Magnetic Resonance spectroscopy
QC	quality control
