# Supplementary material for: Identification and Quantification of a Pneumococcal Cell Wall Polysaccharide by Antibody-Enhanced Chromatography Assay
Source: Vaccines (Basel). 2024 Apr 28;12(5):469. doi: 10.3390/vaccines12050469 (PMC11126027; doi:10.3390/vaccines12050469)
Supplement: Supplementary file 1 [file vaccines-12-00469-s001.zip › vaccines-2937396-supplementary.pdf]

Supplementary Material:

Identification and Quantification of a Pneumococcal Cell Wall Polysaccharide by Antibody Enhanced Chromatography Assay

James Z Deng[1], Zhifeng Chen[4], James Small[2], Yue Yuan[2], Kara Cox[4], Aimin Tang[4], Jeanette Roman[3], Liming Guan[3], Katrina Feller[3], Frances Ansbro[3], Kalpit Vora[4]*

[1]Vaccine Analytical Research & Development, [2]Analytical Enabling Capabilities, [3]Cell Potency Assays, MRL Analytical Research & Development; [4]Infectious Diseases and Vaccines Discovery

Merck & Co., Inc., Rahway, NJ, USA

Table S1. Linearity range data for Figure 4. Peak area was averaged from duplicate injections.

Standard	[C-Ps2] (µg/mL)	FLR peak area
STD-1	0.038	4584243
STD-2	0.077	6470121
STD-3	0.153	12968044
STD-4	0.307	26406878
STD-5	0.460	39588339
STD-6	0.613	50586978
STD-7	0.766	64358442
R^2	0.9992	

Table S2. C-Ps2 quantification precision assessment for 5 PnPs sertypes in six runs. Peak area was averaged from duplicate injections. % C-Ps2 was obtained by Sample [C-Ps2] divided by Sample [Total Ps].

Experiment-1	Sample [Total Ps] (µg/mL)	Dilution	FLR Peak Area	Measured [C-Ps2] (µg/mL)	Sample [C-Ps2] (µg/mL)	% C-Ps2
ST-4	100	40	33980613	0.224	9.0	9.0
ST-6A	100	10	55478804	0.351	3.5	3.5
ST-9V	100	2	24298118	0.167	0.3	0.3
ST-12F	100	20	47823025	0.306	6.1	6.1
ST-19A	100	10	50769460	0.323	3.2	3.2
Standard Curve R^2			0.995828331			
Intercept			-3916297.64			
Slope			169276178.1			

Experiment-2	Sample [Total Ps] (µg/mL)	Dilution	FLR Area	Measured [C-Ps2] (µg/mL)	Sample [C-Ps2] (µg/mL)	% C-Ps2
ST-4	100	40	33850461	0.224	8.9	8.9
ST-6A	100	10	54433169	0.345	3.4	3.4
ST-9V	100	2	24713348	0.170	0.3	0.3
ST-12F	100	20	47679658	0.305	6.1	6.1
ST-19A	100	10	49969650	0.318	3.2	3.2
Standard Curve R^2			0.9980			
Intercept			-4161145.051			
Slope			169962384.3			

Experiment-3	Sample [Total Ps] (µg/mL)	Dilution	FLR Area	Measured [C-Ps2] (µg/mL)	Sample [C-Ps2] (µg/mL)	% C-Ps2
ST-4	100	40	33352699	0.230	9.2	9.2
ST-6A	100	10	53249373	0.351	3.5	3.5
ST-9V	100	2	24103672	0.174	0.3	0.3
ST-12F	100	20	47109572	0.313	6.3	6.3
ST-19A	100	10	48981074	0.325	3.2	3.2
Standard Curve R^2			0.997309788			
Intercept			-4522813.743			
Slope			164723862.7			

Experiment-4	Sample [Total Ps] (µg/mL)	Dilution	FLR Area	Measured [C-Ps2] (µg/mL)	Sample [C-Ps2] (µg/mL)	% C-Ps2
ST-4	100	40	35780760	0.228	9.1	9.1
ST-6A	100	10	49987589	0.306	3.1	3.1
ST-9V	100	2	25219551	0.171	0.3	0.3
ST-12F	100	20	46456145	0.287	5.7	5.7
ST-19A	100	10	65717763	0.391	3.9	3.9
Standard Curve R^2			0.9951			
Intercept			-6200337.989			
Slope			183782469.3			

Experiment-5	Sample [Total Ps] (µg/mL)	Dilution	FLR Area	Measured [C-Ps2] (µg/mL)	Sample [C-Ps2] (µg/mL)	% C-Ps2
ST-4	100	40	36334693	0.210	8.4	8.4
ST-6A	100	10	51612263	0.287	2.9	2.9
ST-9V	100	2	25638340	0.156	0.3	0.3
ST-12F	100	20	50790085	0.283	5.7	5.7
ST-19A	100	10	72922774	0.394	3.9	3.9
Standard Curve R^2			0.996980507			
Intercept			-5187280.18			
Slope			198092138.6			

Experiment-6	Sample [Total Ps] (µg/mL)	Dilution	FLR Area	Measured [C-Ps2] (µg/mL)	Sample [C-Ps2] (µg/mL)	% C-Ps2
ST-4	100	40	36903472	0.222	8.9	8.9
ST-6A	100	10	51580167	0.299	3.0	3.0
ST-9V	100	2	25282217	0.162	0.3	0.3
ST-12F	100	20	48833858	0.284	5.7	5.7
ST-19A	100	10	71283000	0.401	4.0	4.0

Standard Curve R^2	0.995572499			
Intercept	-6015654.775			
Slope	192949534.3			

Table S3. Preparation of spike sample and calculation for spike recovery

1. Sample Preparation

Spike sample	100 µg/mL ST-19A (µL)	mAb (1.0 mg/mL) (µL)	1.53 ug/mL C-Ps2 stock spike in (µL)	Binding buffer (µL)	Total Vol (µL)	Spike dilution	Theoretical spike [C-Ps2] (ug/mL)
Spike-Ctrl	10	15		185	200		
Spike-1	10	15	20	155	200	10	0.153
Spike-2	10	15	40	135	200	5	0.306
Spike-3	10	15	70	105	200	2.86	0.536

2. Spike recovery calculation

Sample	FLR peak area	(Spike-Ctrl) FLR peak area	Measured spike [C-Ps2] (µg/mL)	Theoretical spike [C-Ps2] (µg/mL)	% Rec
19A-Ctrl	14442536				
Spike-1	28113195	13670659	0.159	0.153	104
Spike-2	41702632	27260096	0.322	0.307	105
Spike-3	62493826	48051290	0.572	0.536	107
RSQ			0.999219701		
Intercept			396030.8849		
Slope			83344001.21		

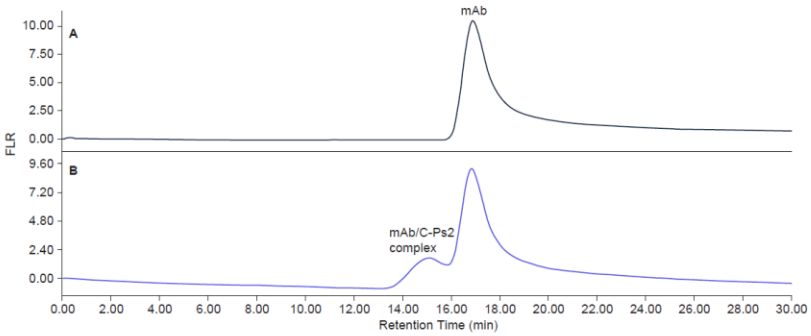

Figure S1. Comparison of mAb binding to SSI CWPS1 (C-Ps1) and mAb binding to SSI CWPS-multi (C-Ps1 + C-Ps2) on AE-HPLC. A. Binding to C-Ps1: no mAb/C-Ps1 complex detected. B. Binding to (C-Ps1 + C-Ps2): mAb/C-Ps2 complex was detected

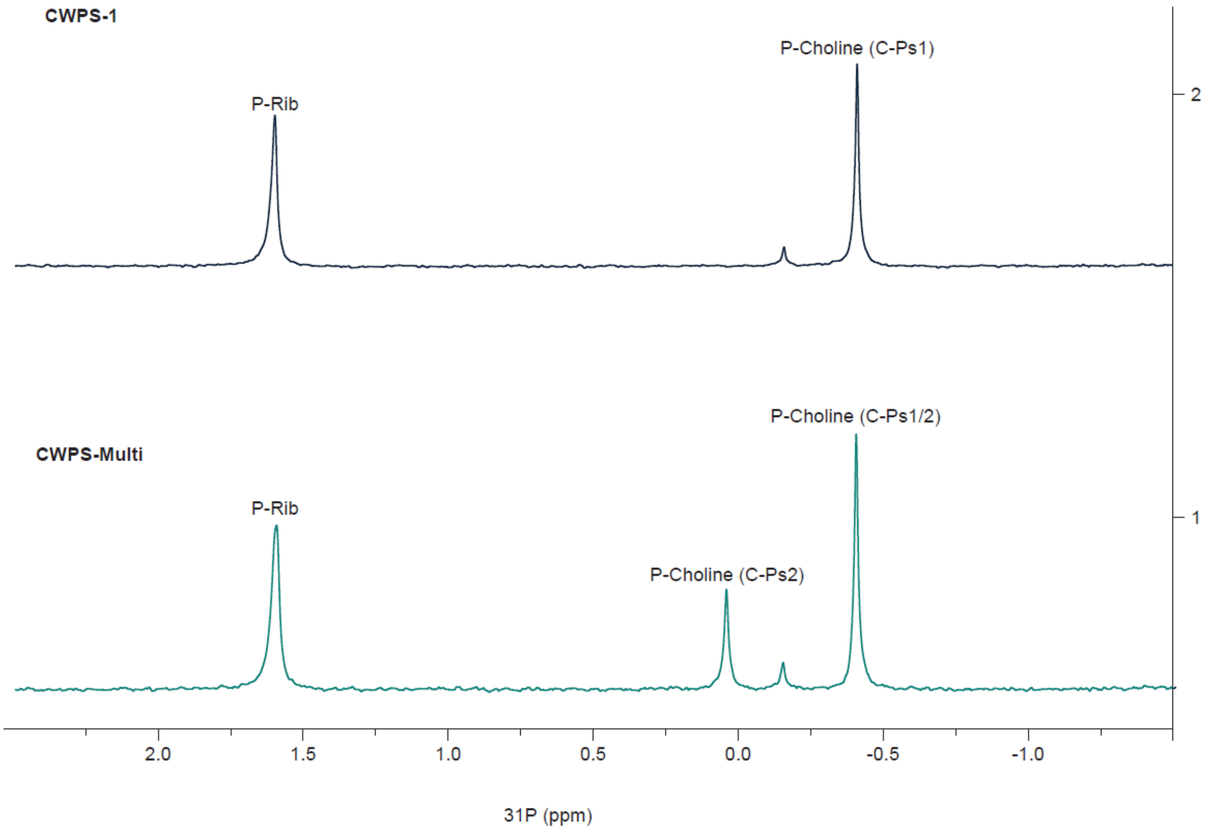

Figure S2. ^{31}P NMR of C-Ps standards: CWPS1 (C-Ps1) and CWPS-multi (C-Ps1 + C-Ps2). One phosphocholine (P-choline) was detected in CWPS1 and two phosphocholines were observed in CWPS-multi.

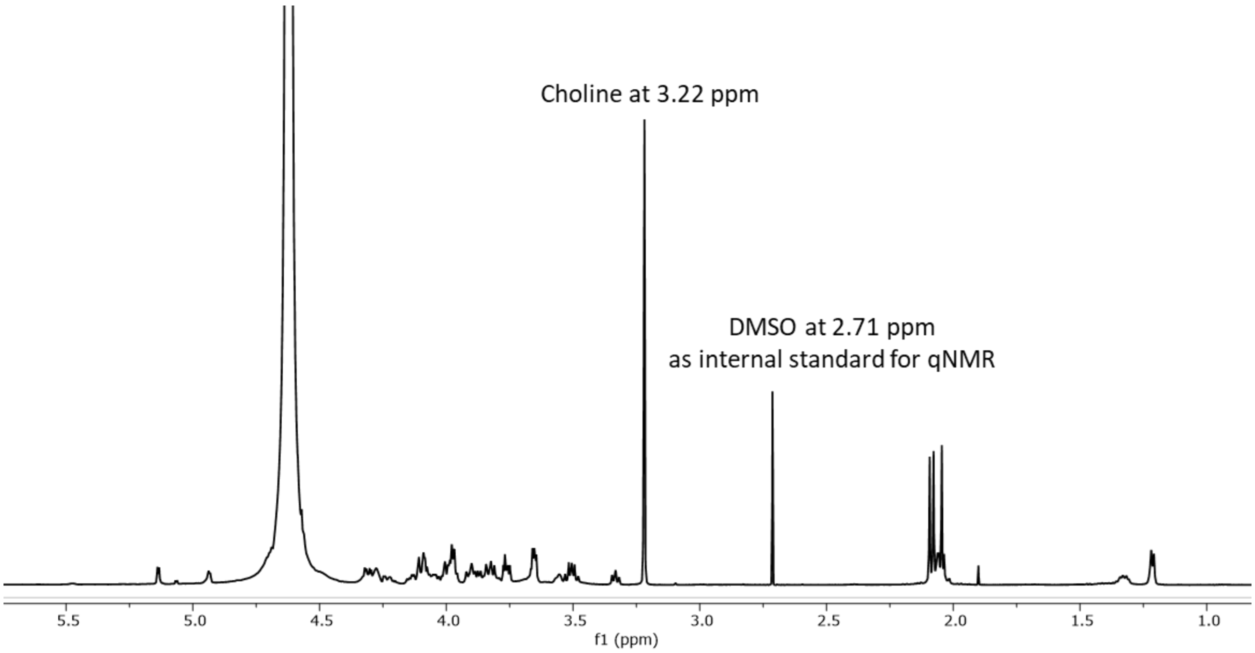

Figure S3. Quantitative NMR analysis for CWPS was performed based on the resonance integration of choline signal at 3.22 ppm and DMSO signal at 2.71 ppm. ^{1}H NMR data were accquired at 49 °C in D_2O. The concentration of DMSO in the solvent is 1.411 mM. Molecular weight (Da) used in the calculations were 1134 for C-Ps1 repeating unit and 1299 for C-Ps2 repeating unit.
